# Origin and occurrence of gem-quality, skarn-hosted barite from Jebel Ouichane near Nador in Morocco

**DOI:** 10.1038/s41598-021-89692-5

**Published:** 2021-05-13

**Authors:** Magdalena Dumańska-Słowik, Beata Naglik, Tomasz Toboła, Tomasz Powolny, Miłosz Huber, Stanislava Milovska, Natalia Dobosz, Kamil Guzik, Aleksandra Wesełucha-Birczyńska

**Affiliations:** 1 Faculty of Geology, Geophysics, and Environmental Protection, AGH-University of Science and Technology, 30 Mickiewicz Av, 30-059 Kraków, Poland; 2 Polish Geological Institute-National Research Institute, Upper Silesian Branch, Królowa Jadwiga str., 41-200 Sosnowiec, Poland; 3 Department of Geology, Soil Science and Geoinformacy, Faculty of Earth Science and Spatial Management, Maria Curie – Skłodowska University, 2d/107 Kraśnickie Rd, 20-718 Lublin, Poland; 4 Earth Science Institute, Slovak Academy of Sciences, 1 Ďumbierska Str, 974 11 Banská Bystrica, Slovakia; 5 Geotech, 1B Chemików Str, 32-600 Oświęcim, Poland; 6 Faculty of Chemistry, Jagiellonian University, 2 Gronostajowa Str., 30-387 Kraków, Poland

**Keywords:** Environmental sciences, Solid Earth sciences

## Abstract

Light-blue barite from Jebel Ouichane in Morocco forms blade-like tabular crystals (up to ca. 10 cm) with superb transparency and lustre and represents one of the most spectacular gem-quality worldwide. The barite is hosted by iron-ore-bearing skarns, developed within Jurassic-Cretaceous limestones, and occurs in close spatial association with calcite. The crystals have their cores enriched in Sr and contain abundant monophase (liquid) fluid inclusions of primary and pseudosecondary origin. The barite probably precipitated slowly at a relatively low supersaturation and under the control of a surface reaction precipitation mechanism. However, there were some episodes during its formation with a fast growth rate and the coupled dissolution and recrystallization processes. A combination of fluid inclusion data and stable *δ*^18^O value for barite (+ 6.71‰ VSMOW) suggests that low-salinity barite-forming solutions resulted from the mixing of strongly-diluted meteoric waters (enriched in light oxygen isotope) with magmatic-hydrothermal fluids under low-temperature conditions (< 100 °C). Meanwhile, the mineralizing fluids must have been enriched in Ba, Sr, Ca, Mg, and other elements derived from the alteration of carbonate and silicate minerals in sedimentary and igneous rocks. The coupling between sulphur and oxygen isotope data (+ 16.39‰ VCDT and + 6.71‰ VSMOW, respectively) further suggests that barite crystallized in steam-heated environment, where SO_4_^2-^ derived from magmatic-hydrothermal SO_2_ reacted with sulphates that originate from the oxidation of H_2_S under near-surface conditions.

## Introduction

Barite (BaSO_4_) is ubiquitously distributed in various geological settings since it not only forms over a large range of pressures and temperatures (1–2000 bar, up to ca. 400 °C), but also shows limited susceptibility to weathering and/or secondary alterations. Hence, this mineral may be hosted by sedimentary, igneous, and metamorphic rocks^1^. In sedimentary sequences, it is common as massive beds, laminations, rosettes, and/or nodules^2^. Overall, four genetic types of barite, marked by variable Sr and S isotopic signatures^3^, in modern marine geological environments can be distinguished^1,4,5^: (1) pelagic/marine, (2) cold-seeps, (3) hydrothermal, and (4) diagenetic. The crystallization of the former (1) is triggered by the decomposition of organic material that leads to the introduction of micro-environments in the water column supersaturated in relation to barite^3^. (2) Cold seeps barite crystallizes at the sediment–water interface and occurs within both active and passive continental margins, whilst its origin is connected with expulsion and overpressure of Ba-rich fluids via tectonic and/or hydrological processes. (3) Hydrothermal barite is associated with submarine volcanic activity but maybe also related to the mixing of late-stage hydrothermal fluids and meteoric waters^3,5,6^. (4) Diagenetic barite crystallizes from pore waters at redox boundaries within sediments. Its formation involves dissolution and reprecipitation of pre-existing barite, followed by sulphate reduction. Finally, some barite deposits may form from the alteration of pre-existing evaporites containing gypsum and anhydrite^7,8^. The temperature of barite formation has been constrained by i.e. Raman carbonaceous material geothermometry^9^, fluid inclusion data^10,11^, and isotopic fractionation between coexisting pyrite-barite pairs^12^. Finally, barite represents a gangue mineral associated with sedimentary-exhalative (SEDEX-type)^13^ and massive sulphide (VMS) deposits^14^, as well as various stratiform and vein ore bodies^15^.

Morocco is one of the leading barite producers in the world. Most barite deposits in this region were emplaced during the rift stage assigned to the Triassic-Jurassic period^16^. The Moroccan production is mainly supported by three vein-type barite deposits, i.e. Ibel Irhoud, Zelmou, Ht. Seksouaa, Drâa-Tafilalet, as well as widespread small outcrops, frequently exploited by local artisanal miners looking for gem-quality specimens^16–18^.

The barite from the Nador region in Morocco flooded the mineral and gem market in the spring of 2012 at the Sainte Marie Aux Mines show and quickly became very popular on the international mineral fairs and exhibitions^19^. It produces some of the most prominent specimens in the world based on the size of particular crystals (up to 10 cm), their light blue coloration contrasting with the surrounding rocks, attractive blade-like habits, as well as superb transparency and lustre. The barite specimens occur in pockets and open fractures in Jurassic-Cretaceous metasediments exposed in the quarries near Nador in northern Morocco (Sidi Lahcen and Ouichane mines). From 2012 till 2015 the quarry's walls were exploited intensively in search of barite^19^.

In this study, we aimed to reconstruct the palaeoenvironmental setting (i.e. origin, pathways of mineralizing fluid, and variations in physicochemical conditions during mineral-forming processes), which prevailed during the crystallization of unique and gem-quality, blue barite from Jebel Ouichane quarry in Nador (Morocco). Our constraints are based on fluid inclusions, isotopic (S, O, and C) composition obtained for both barite and co-paragenetic calcite, supported by the Raman micro-spectroscopy combined with electron-microbe (EMPA) and X-ray fluorescence (XRF).

## Geological setting

**Figure 1:**
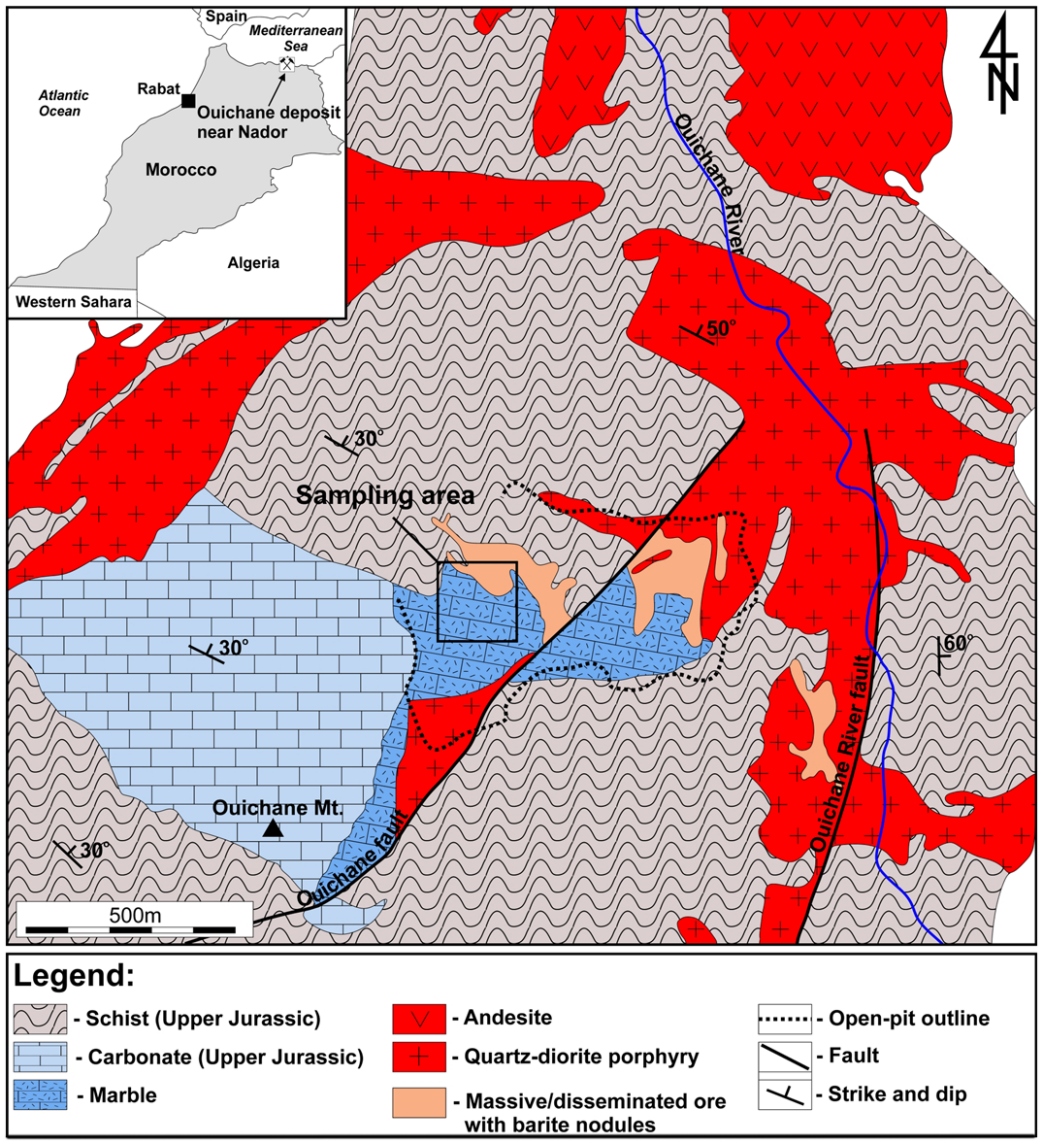
Geological map showing main lithologies found in the vicinity of Jebel Quichane area, modified after Bouabdellah et al. ^21^.

The Jebel Ouichane area, found near Nador city in the north of Morocco, comprises the exposures of Jurassic limestones with an admixture of clay sediments and subordinate igneous and metamorphic rocks (Fig. 1)^20,21^. Barite nodules occur in a closed quarry complex, which is situated on the slope of the Ouichane Mountain and represented an important source of iron ore to many countries of western Europe during the first half of the twentieth century. The mineralization, mainly in the form of magnetite, hematite, limonite, pyrite, and rarely chalcopyrite and pyrrhotite, has likely resulted from the contact skarn-type metamorphism of the Jurassic limestone by Miocene quartz diorite porphyry intrusion^19^. As noted by Bouabdellah et al.^21^, the timing of mineralization (7.04 ± 0.47 Ma) well corresponds to the crystallization age of the Ouichane quartz-diorite porphyry (7.58 ± 0.03 Ma), hence providing a strong evidence for the genetic relationship between ore mineralization and Late Neogene magmatism.

As a result of metasomatism, skarn-type ore deposits with high contents of iron, coupled with low sulphur amounts, were mined from Jurassic-Cretaceous metasediments. However, with the increased demand, and thus the deepening of the ore extraction range, it was noted that the proportion of pyrite to magnetite increased with depth. This had implied additional desulphurization costs and reduced the profitability of pre-exploration. As a consequence, the mine was closed in 1950^20^.

In the vicinity of Ouichane mountain, Jurassic limestone dips 30° from the top towards the north to the exploited iron deposit. The limestone outcrops in the form of large beds with a maximum thickness of 250 m, being slightly converted into marble in that region. In the north-western part of the mine, the pure and partially metamorphosed limestone gradually changes into a series of clay rocks. The stratification of that sediments is quite complicated due to tectonic activity recognized in this region, indicated by the series of hollows, faults, micro-cracks, etc.^20,21^.

In the valleys, north of the deposit in the Ouichane mountain, the Jurassic-Cretaceous limestone is overlayered by a series of post-orogenic terrigenous deposits including yellow sandy clays, marls, and conglomerates assigned to the early Tertiary period. Both limestones and terrigenous deposits are covered by tuffs and biotite andesites originating from the former Gourougou stratovolcano found to the west of Melilla and other smaller adjacent craters in that region^20^. Igneous rocks from the study area are chiefly represented by granitic to dioritic rocks containing many argillite "xenoliths". Quartz and plagioclase aplites, microdiorites, and porphyry micromonzonites are also abundant in this area^20,21^. The contact metamorphism of limestone and argillite is negligible, often limited to the recrystallization zone, i.e. < 1 m within limestone and less than a few cm from the intrusion^20^.

## Results

**Figure 2:**
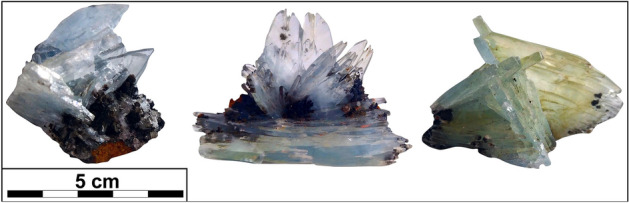
An example of gem-quality specimens of blue barite from Jebel Ouichane.

Barite forms well-crystallized, bladed-tabular, light-blue crystals with an average size of 3–4 cm, locally reaching up to 10 cm. The thin individual plates/blades are frequently arranged parallel to each other and grouped in characteristic aggregates (Fig. 2). Rarely, they were found in either radiating bundles or euhedral tabular crystals that project into open vugs. The crystals of barite are commonly embedded within host ore-bearing skarn-type deposits enriched in iron-bearing phases. Occasionally, they occur on a matrix of white–grey calcite.

### Petrography of barite bearing rock

**Figure 3:**
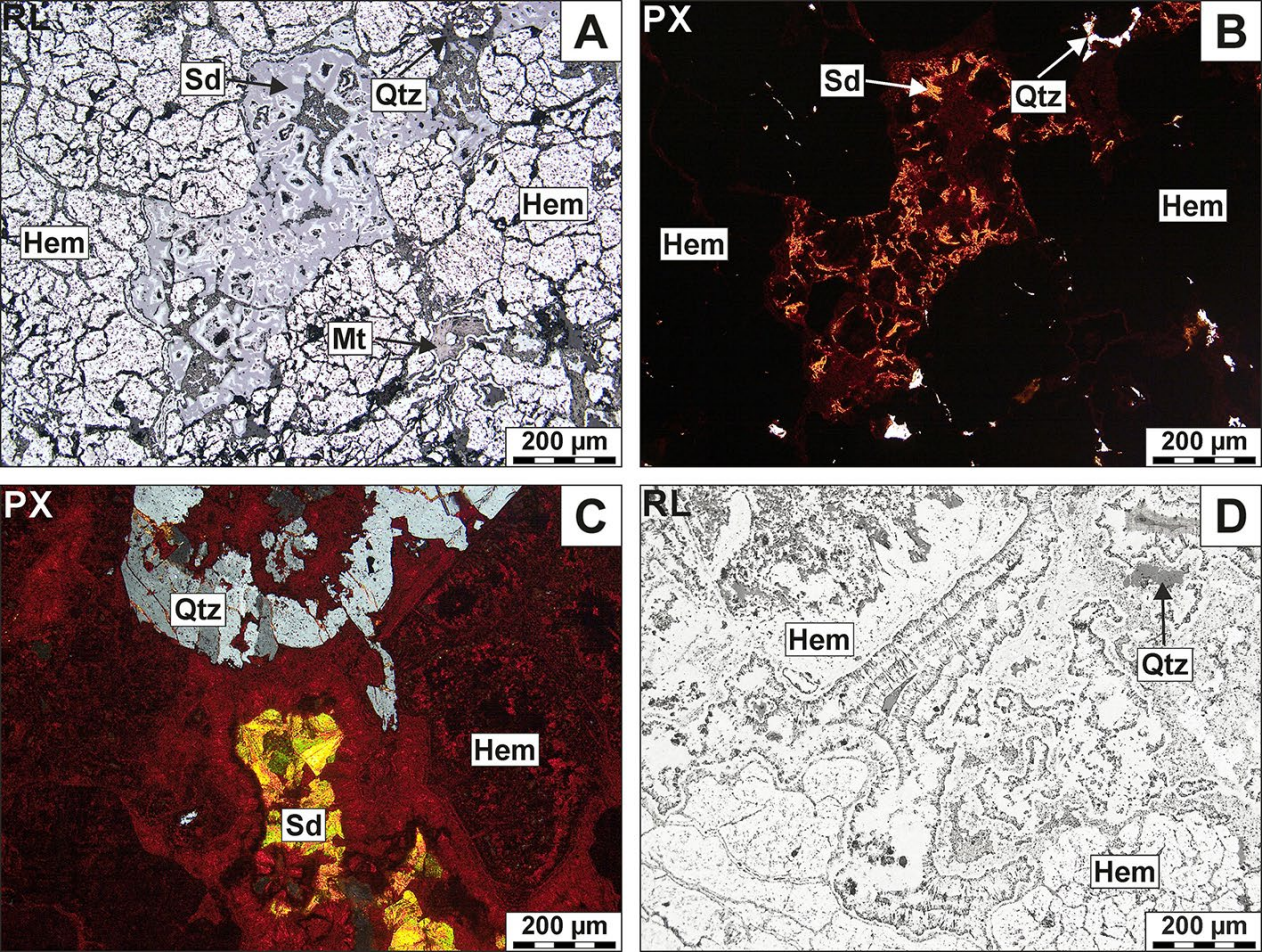
(**A**, **B**) Intergrowths made of siderite (Sd) and hematite (Hem) (central part of the image), surrounded by replacive isometric crystals of hematite (martite) formed after magnetite (Mt); (**C**, **D**) Colloform aggregates of hematite accompanied by minor siderite and quartz (Qtz); Note that PX denotes transmitted light mode (with crossed polars) and RL corresponds to the reflected-light mode.

The barite host rock is mainly composed of Fe oxides/hydroxides such as hematite, goethite, magnetite, and subordinate carbonates, i.e. calcite and siderite. Hematite is the major component of the rocks while its formation is linked to various stages of martitization (Fig. 3A,B). This process proceeded along the edges of crystal faces, from the rim toward the core of the magnetite grains. As a result, newly formed hematite usually shows mesh or colloform microtextures (Fig. 3C,D). Calcite occurs as rhombohedral crystals with characteristic polysynthetic twinning, or with siderite that forms small aggregates or fills thin veins intersecting the rock. Mn-oxides, chalcopyrite, and pyrite are accessory phases of the rock.

### Fluid inclusions data

**Figure 4:**
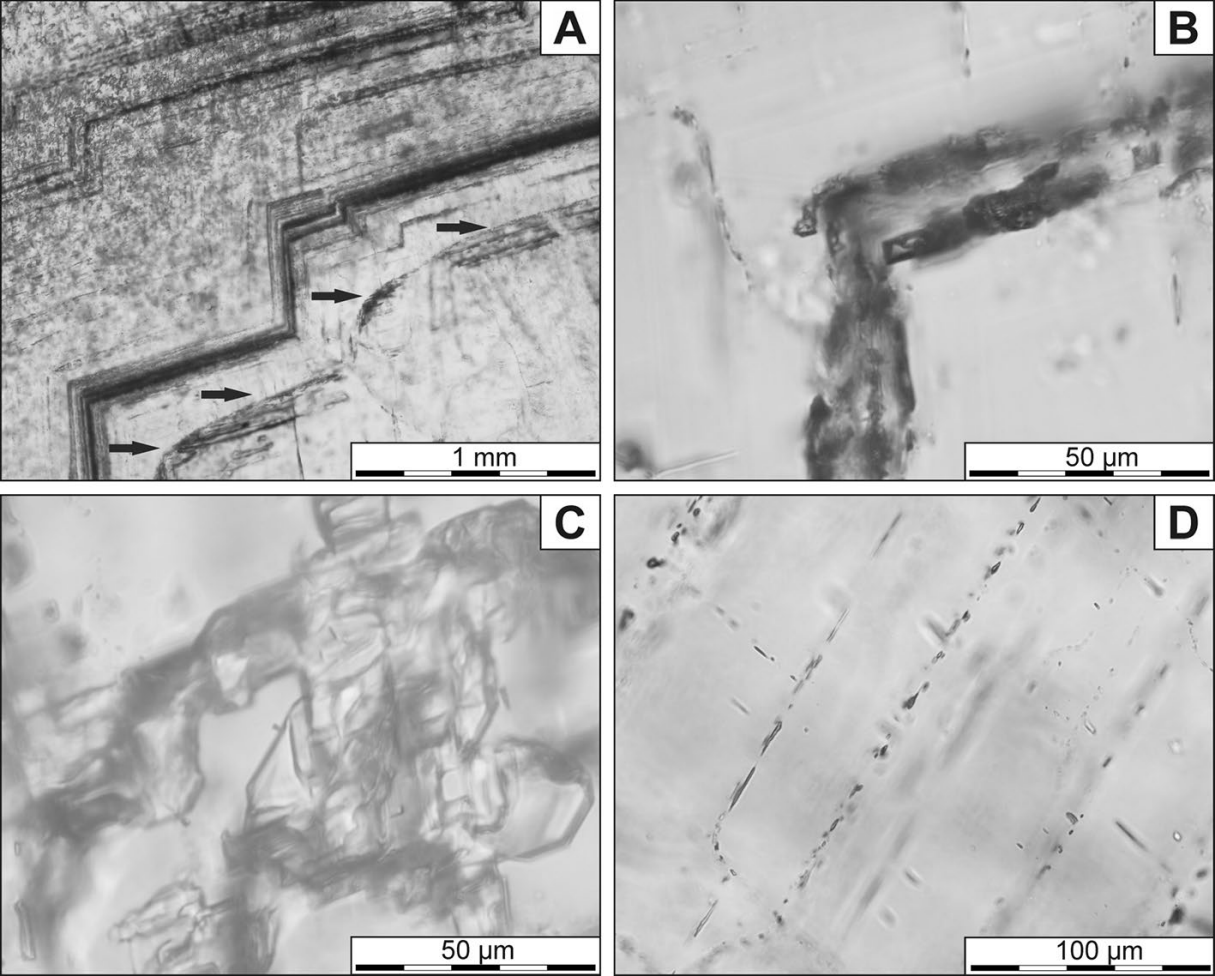
Fluid inclusions assemblages in barite crystals: (**A**) Arrangement of the first and second type of primary FIAs with densely packet inclusions (dark belts). Arrows show the second type of FIA emphasizing the presence of a blurred surface. (**B**) The inclusions in the first type of FIA. (**C**) The inclusions in the second type of FIA. (**D**) Arrangement of the third type of FIAs of primary origin.

Barite crystals host an abundance of fluid inclusions assemblages (FIAs) in both parallel and perpendicular sections. In the respect of their manner of occurrence (size, shape, position, etc.), six types of FIAs were distinguished. The first type (1) occurs only in some places and form opaque belts along the growth planes of barite crystals (Fig. 4A). Such a position indicates the primary origin of these inclusions. Furthermore, these FIAs are composed of densely packed one-phase, liquid inclusions, of ~ 50- ~ 200 µm in length and with rectangular, tubular, or slightly irregular shapes (Fig. 4B). In some cases, where their course changes at right angles, these FIAs partly disappear and are replaced by the second type of FIAs (2), which forms surfaces or belts with an arcuate course (Fig. 4A). They (2nd type) are also composed of densely packed, one-phase (liquid) inclusions. Their shape is more irregular, whilist the size reaches up to 200 µm (Fig. 4C). The inclusions are frequently interconnected and arranged in the form of nets. This type of FIAs should be considered as primary originated, which marked the blurred (dissoluted) surfaces formed as a result of physicochemical changes of crystallization conditions.

**Figure 5:**
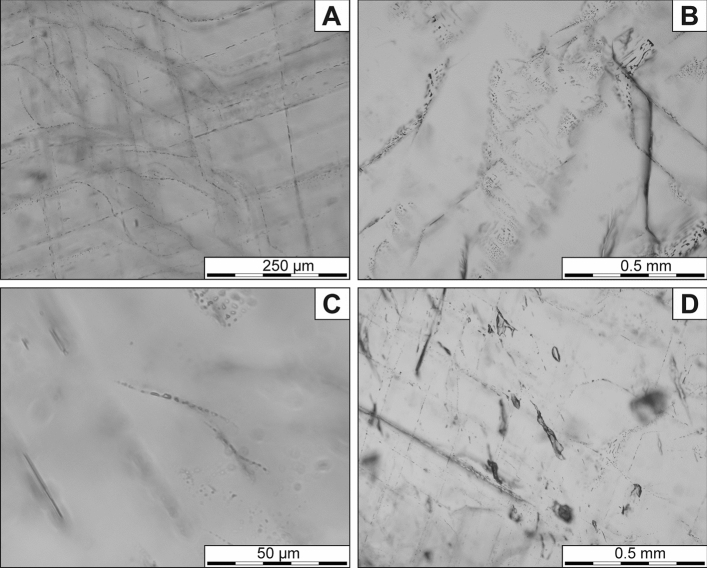
Fluid inclusions assemblages in barite crystals: (**A**) Areas with the blurred course of the third type of FIAs. (**B**) The fourth type of FIAs with pseudosecondary inclusions. (**C**) S-shaped FIAs of the fifth type with pseudosecondary inclusions. (**D**) Large, secondary inclusions of the sixth FIA type.

The third type of FIAs (3) is grouped in linear planes and arranged parallel to the both cleavage and growth planes of the host mineral (Fig. 4D). The course of the FIAs does not cover whole crystals. At the ends of their course, the inclusions gradually become smaller and eventually disappear. Generally, inclusions in these FIAs are tubular or lenticular, often flattened with oval shapes or show tails, which indicate the necking down process^22^. They reach up to 10 µm in size. In some parts of the crystal, their linear course is confused, becomes curved or wavy until they disappear completely (Fig. 5A). These confused areas cover only small parts of barite crystals. Due to FIAs position in the crystals, they should be considered as pseudosecondary.

The fourth type of inclusions (4) forms elongated areas, with their course oblique to the crystal growth zones (Fig. 5B). These areas are limited by the curved surfaces of FIAs. In these zones, the fluid inclusion assemblages are distributed along the cleavage planes and occur as straight planes. The inclusions size ranges from a few up to ~ 10 µm and their shape varies from oval to tubular. They often show tails, which indicate the necking down process^22^. As in the third type, the areas of their occurrence cover only small parts of barite crystals and therefore they should be considered as pseudosecondary-originated.

The fifth type of FIAs (5) forms short curved or sigmoid lines (Fig. 5C). They are composed of rectangle-shaped, slightly elongated, one-phase (liquid) inclusions. Their size reaches up to ~ 10 µm in the centre of the FIA, whereas at the ends of their course, the inclusions gradually become smaller until they eventually disappear. Their form of occurrence indicates a pseudosecondary origin.

In some parts of the crystals larger inclusions reached up to ~ 200 µm in length (the sixth FIAs) also occur (Fig. 5D). Their shape is irregular, sometimes flatten or oval and the inclusions are one-phase (liquid). They commonly exhibit characteristic tails triggered by the necking down process. Their longer axes are inclined to the cleavage planes, which may indicate their secondary nature.

The attempts to nucleate vapour bubbles (see Methods section) in all one-phase inclusions have failed. Even at a temperature of around 0 ºC the vapour bubble did not nucleate. Therefore, it was impossible to measure the homogenization temperatures. The lack of water vapour nucleation may indicate, that the molar volume of solution in inclusions is approximately 18 cm^3^/mol or lower if clean water is considered ^23^.

**Figure 6:**
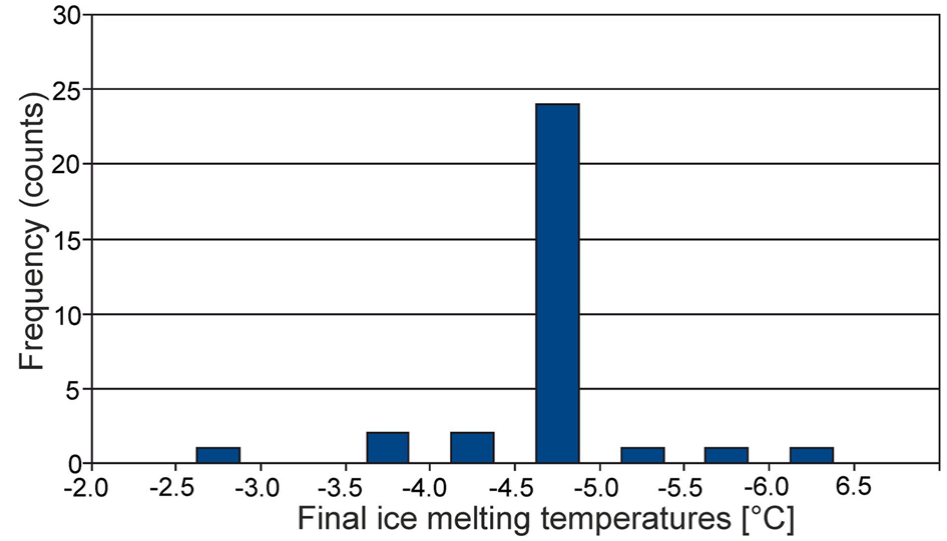
Histogram of the ice melting temperature in primary inclusions.

The low-temperature measurements aimed at determining the salinity of the inclusions show that the last ice melting temperatures in the primary FIAs are in the range − 2.5 to − 6.2 °C which corresponds to salinity 4.18–9.34 wt% NaCl Eq. ^24^. Most of the ice melting temperatures is in the range − 4.5 to − 5.0 (Fig. 6) which corresponds to salinity 7.17–7.86 wt% NaCl eq.

### X-ray fluorescence

**Figure 7:**
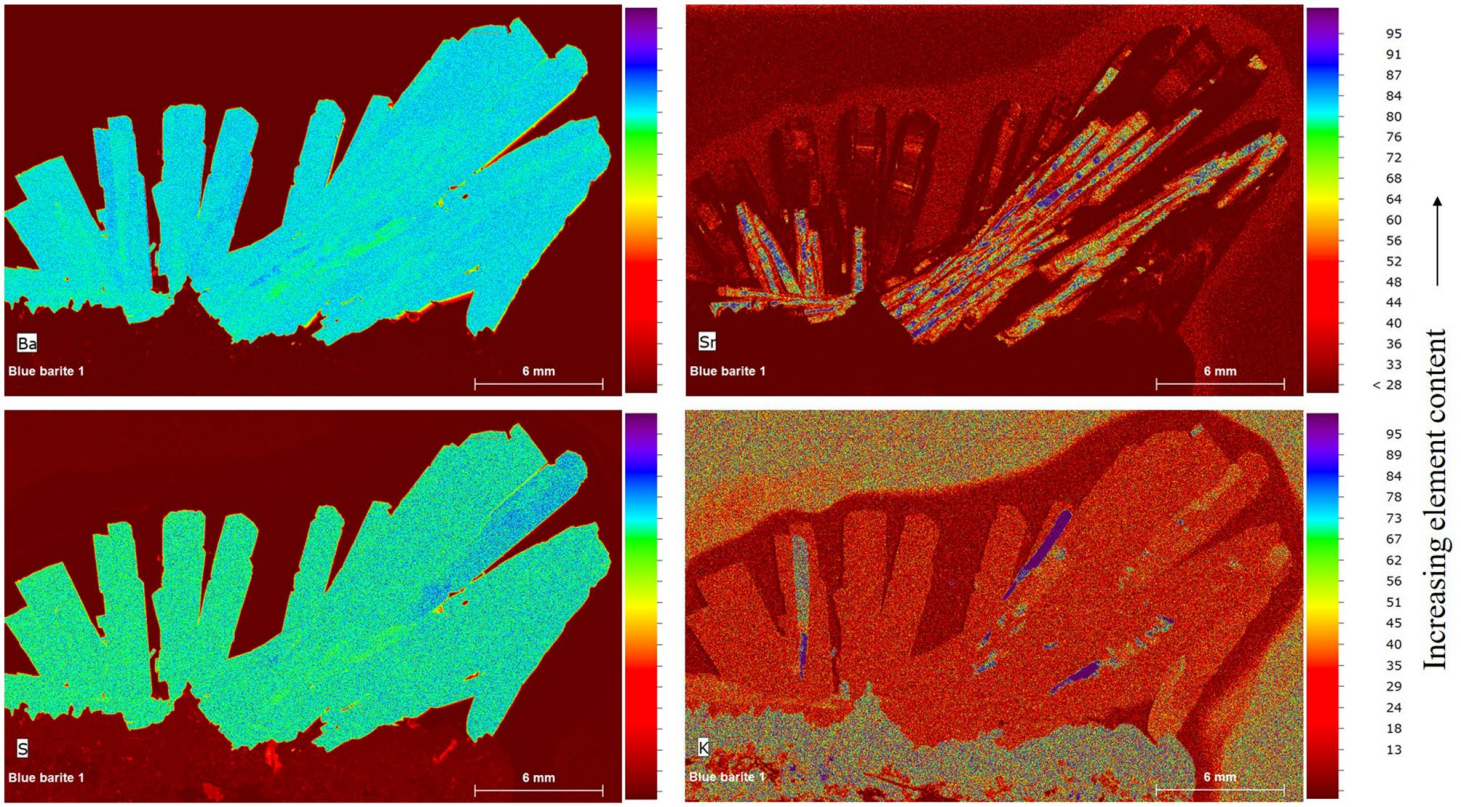
False-colour elemental distribution maps for Ba, S, Sr, and K obtained by the Energy-dispersive μX-ray Fluorescence Spectrometry of barite crystals (polished section).

Semi-quantitative chemical analysis (Table 1) and elemental areal mapping of barite crystal aggregate show enrichment in Ca, Cu, K, Fe, Sr, and Zn. Indistinctive sectorial zoning of Ba and Sr distribution was observed (Fig. 7). An elevated concentration of Sr and K was found only in specific crystal zones; Sr particularly accumulates in the core of the crystals.

**Table 1:** Chemical composition of barite in weight % (wt%), measured by Energy-dispersive μX-ray Fluorescence Spectrometry, recalculated to fixed SO_3_ content and a total of 100 (wt%). Al_2_O_3_, MgO, MnO, and Na_2_O contents were below the detection limit. Error is 0.00 for all (in wt. %, 1 Sigma).

Oxide content (wt. %)	BaO	CaO	CuO	FeO	K_2_O	SO_3_	SrO	ZnO	Total
Analysis									
1	61.59	0.33	0.00	0.00	0.00	34.30	1.00	0.04	100.00
2	59.43	0.23	0.02	0.00	0.44	34.30	0.41	0.18	100.00
3	63.46	0.26	0.07	0.02	0.48	34.30	0.31	0.08	100.00
4	63.09	0.00	0.05	0.07	2.39	34.30	0.91	0.04	100.00
5	63.08	0.00	0.18	0.23	0.18	34.30	0.63	0.00	100.00
6	65.02	0.05	0.06	0.00	0.29	34.30	0.06	0.08	100.00
7	59.29	0.00	0.10	0.00	0.00	34.30	0.51	0.16	100.00
8	62.36	0.09	0.29	0.01	0.72	34.30	0.00	0.06	100.00
9	64.75	0.33	0.05	0.19	0.82	34.30	0.79	0.00	100.00
10	64.73	0.00	0.28	0.00	0.30	34.30	0.41	0.08	100.00
11	59.86	0.00	0.07	0.07	0.00	34.30	0.29	0.08	100.00
12	60.93	0.00	0.18	0.26	0.00	34.30	0.75	0.03	100.00
13	56.74	0.00	0.10	0.19	1.13	34.30	1.06	0.19	100.00
14	62.22	0.00	0.15	0.00	0.81	34.30	0.23	0.18	100.00
15	65.08	0.24	0.03	0.00	0.00	34.30	0.24	0.08	100.00
Minimum	56.74	0.05	0.02	0.01	0.18	34.30	0.06	0.03	100.00
Maximum	65.08	0.33	0.29	0.26	2.39	34.30	1.06	0.19	100.00
Average	62.11	0.22	0.12	0.13	0.76	34.30	0.54	0.10	100.00

### Electron microprobe analyses (EMPA)

**Figure 8:**
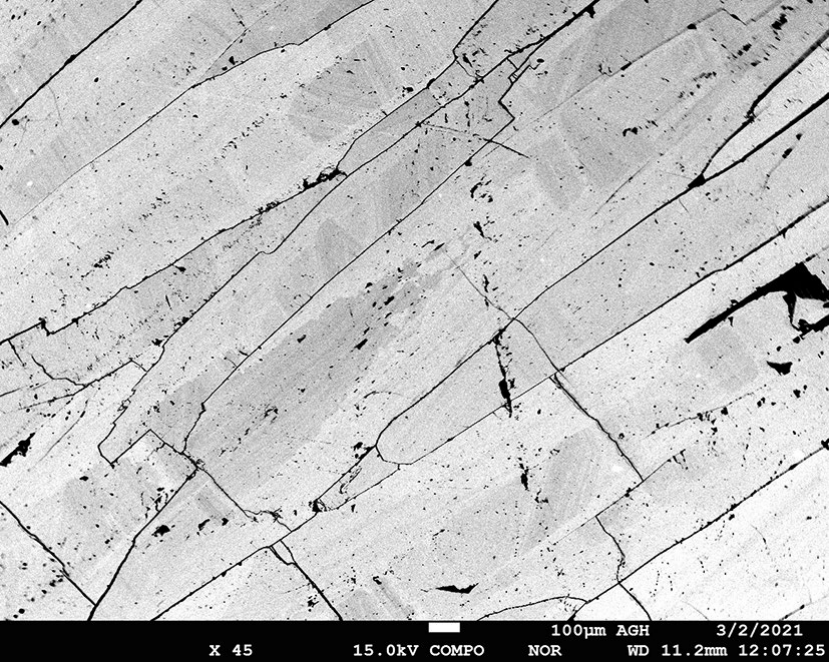
BSE image of barite crystal showing chemical zonation.

Blue barite from Nador has a simple composition (Table 2) as it contains 60.09–66.01 wt% of BaO and 34.63–35.30 wt% of SO_3_. Ba is locally substituted by Sr (0.00–2.59 wt% SrO), Na (0.00–0.11 wt% Na_2_O), Al (0.03–0.13 wt% Al_2_O_3_) and Ca (0.00–0.09 wt% CaO) in the crystal cores. As a result, under BSE imaging barite shows distinct zoning (Fig. 8). The darker patches are more enriched in Sr relative to lighter patches in the crystals. Strong negative correlations were observed between SrO *vs* BaO (R = − 0.97) and [SrO + CaO] *vs* BaO (R = − 0.98), consistent with Sr replacing Ba.

**Table 2:** Representative electron microprobe data of barite from Jebel Ouichane.

Spot no	2	3	4	5	6	7	8	9	10	11	12	13	14	15	16	17
	Wt.%															
Al_2_O_3_	0.12	0.13	0.10	0.11	0.10	0.08	0.12	0.10	0.07	0.04	0.05	0.04	0.07	0.06	0.05	0.03
CaO	0.05	0.00	0.00	0.00	0.00	0.03	0.03	0.00	0.00	0.00	0.09	0.03	0.00	0.00	0.00	0.00
Na_2_O	0.11	0.09	0.00	0.00	0.00	0.00	0.00	0.00	0.00	0.06	0.00	0.00	0.00	0.00	0.00	0.11
BaO	65.90	64.97	64.82	65.56	65.64	65.35	64.77	65.81	64.56	60.09	64.68	63.42	64.05	64.46	65.92	66.01
SrO	0.20	0.61	0.53	0.35	0.39	0.28	0.53	0.28	0.71	2.59	0.54	1.22	1.05	0.65	0.00	0.21
SO_3_	35.13	35.27	35.25	35.08	34.98	35.10	34.63	35.06	35.20	35.15	34.83	35.24	35.30	34.77	35.26	35.09
Total	101.52	101.06	100.71	101.10	101.11	100.83	100.09	101.24	100.55	97.93	100.18	99.96	100.47	99.94	101.23	101.43
	Cations per formula unit [apfu]															
Al	0.006	0.006	0.004	0.005	0.004	0.004	0.006	0.004	0.003	0.002	0.002	0.002	0.003	0.003	0.002	0.001
Ca	0.002	0.000	0.000	0.000	0.000	0.001	0.001	0.000	0.000	0.000	0.004	0.001	0.000	0.000	0.000	0.000
Na	0.008	0.006	0.000	0.000	0.000	0.000	0.000	0.000	0.000	0.005	0.000	0.000	0.000	0.000	0.000	0.008
Ba	0.980	0.965	0.965	0.978	0.981	0.976	0.977	0.982	0.963	0.903	0.973	0.947	0.953	0.971	0.981	0.984
Sr	0.004	0.013	0.012	0.008	0.009	0.006	0.012	0.006	0.016	0.058	0.012	0.027	0.023	0.015	0.000	0.005
S	1.000	1.003	1.005	1.002	1.001	1.004	1.001	1.002	1.005	1.011	1.003	1.007	1.006	1.003	1.005	1.002

### Raman micro-spectroscopy

**Figure 9:**
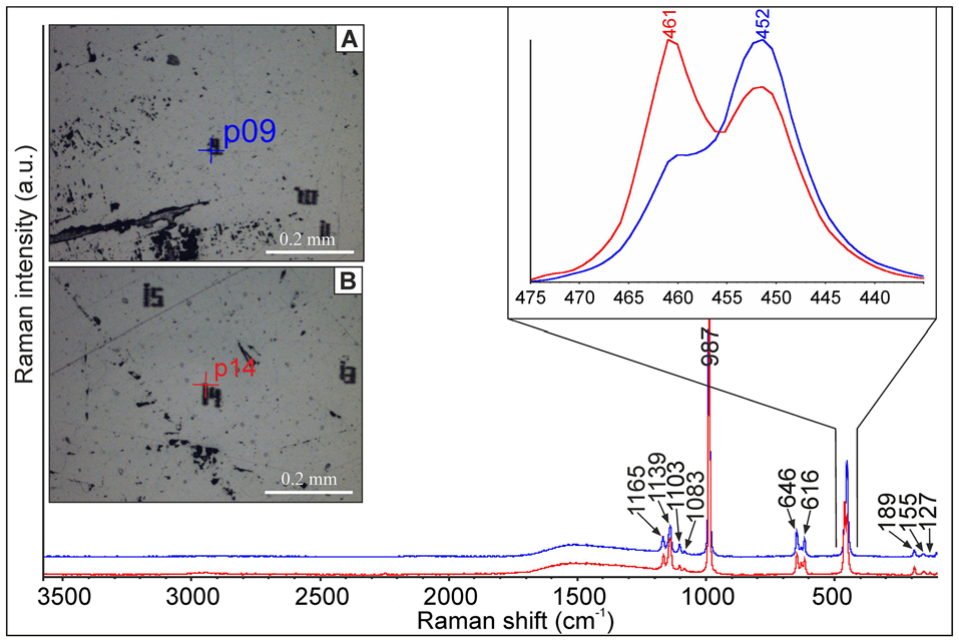
Microphotos and Raman spectra collected in the various regions of barite crystal: red and blue lines correspond to spectra collected in microprobe analytical points 14 and 9, respectively (vide Table 2). The inset shows the variations in the intensity of the Raman bands at 461 and 452 cm^−1^ recorded for crystal domains slightly differing in Sr and Ba content.

The Raman spectrum of barite (Fig. 9) consists of an intense ν_1_ band, which corresponds to the nondegenerate symmetric stretching of SO_4_ tetrahedra at^25^ 987 cm^−1^. The other characteristic bands of SO_4_ ν_2_ , ν_3_ and ν_4_ , were found at: ν_2_—452 cm^−1^ and 461 cm^−1^; ν_3_—1083 cm^−1^, 1139 cm^−1^ and 1165 cm^−1^; ν_4_—616 cm^−1^ and 646 cm^−1^ (Table 3). They arise from double degenerate, symmetric bending (ν_2_), triple degenerate asymmetric stretching (ν_3_), and triple degenerate asymmetric bending (ν_4_) vibrations^25,26^. The extra low-intensity band at 1103 cm^−1^ could be attributed to the ν_3_ mode in the sulphate^27^. The less intensive bands below 400 cm^−1^ (127, 155, and 189 cm^−1^) are assigned to the vibration of the Ba–O bonds^25^.

**Table 3:** The Raman vibrational modes in barite.

Raman-active bands in barite (cm^−1^)	Mode assignment^28,29^
127	M–O
155	M–O
189	M–O
452	ν_2_ SO_4_
461	ν_2_ SO_4_
616	ν_4_ SO_4_
646	ν_4_ SO_4_
987	ν_1_ SO_4_
1083	ν_3_ SO_4_
1139	ν_3_ SO_4_
1165	ν_3_ SO_4_

The coupling between Raman spectroscopic studies and EMPA data revealed that positions of diagnostic Raman bands have mostly remained unaffected by the variations of the main element composition of barite. Only slight variations were observed in the lower range of Raman shift, i.e. 400–500 cm^−1^: variable proportions of the peak heights at 461 cm^−1^ and 452 cm^−1^ were noted (Fig. 9 inset). The ratio of the height of both peaks (H_451_/H_461_) and the SrO content (wt.%) in the whole population of analytical points shows no correlation.

**Figure 10:**
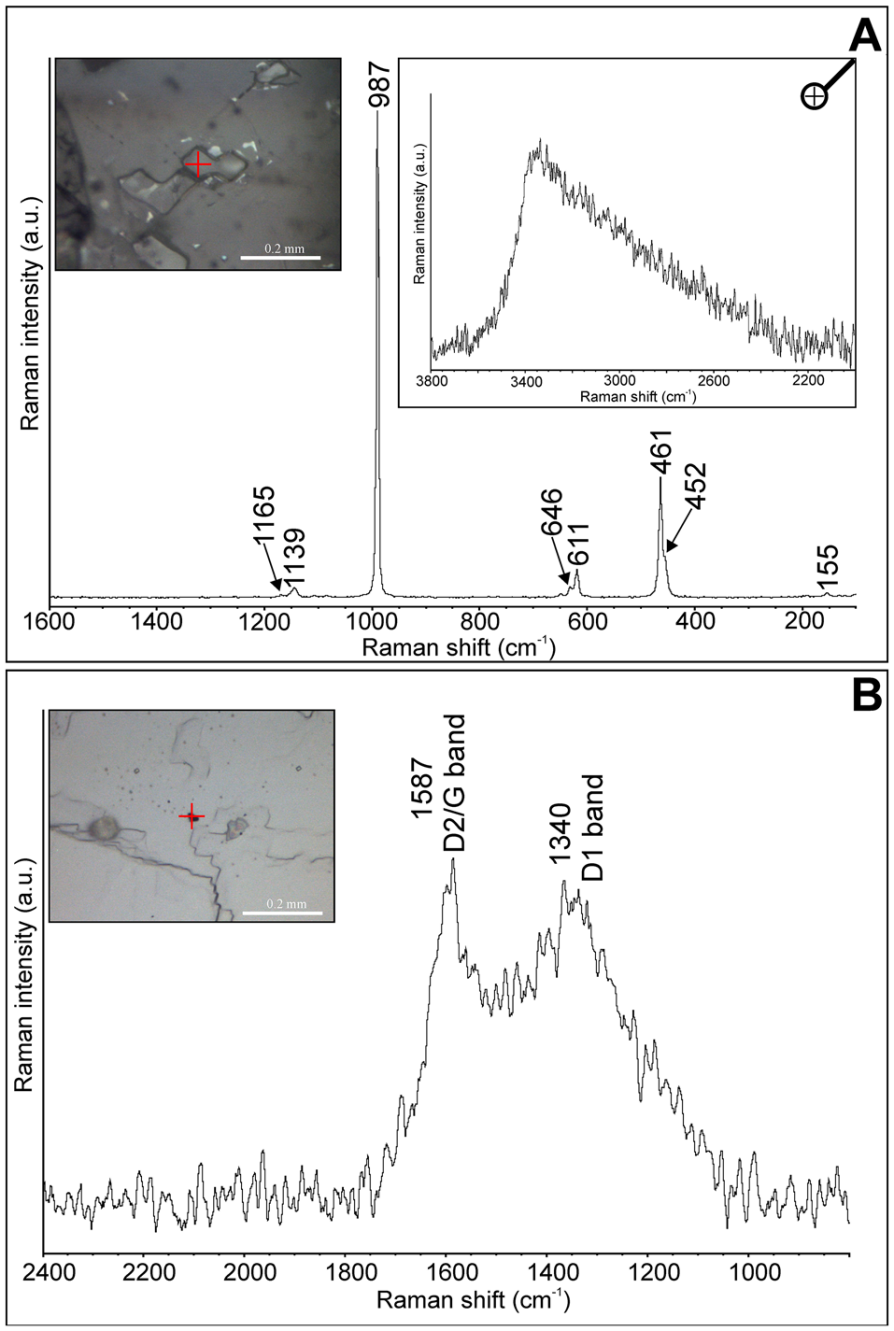
Microphotos and Raman spectra collected for: (**A**) fluid inclusions in barite. Note that most bands correspond to the host mineral; the presence of water in fluid inclusions is indicated by a broad band about 3300 cm^−1^ (see inset); (**B**) small inclusions of organic matter; note that D band refers to disordered carbon, whereas G bands is related to in-plane stretching vibrations of between sp2 carbon atoms.

Raman spectra collected from fluid inclusions revealed the presence of water, as evidence by a broad asymmetric band found in the region 3800–2000 cm^−1^ with a maximum of ~ 3300 cm^−1^ (Fig. 10A). These observations infer that FIAs are chiefly composed of water solutions. Rare, small inclusions of carbonaceous matter, indicated by two broad bands at 1587 cm^−1^ (D2/G band) and 1340 cm^−1^ (D1 band)^30^, are found within barite. The broadening of carbon-related Raman bands (Fig. 10B), as well as their position further suggest that carbonaceous matter shows a low degree of its maturity, typical of amorphous carbon^31^.

**Figure 11:**
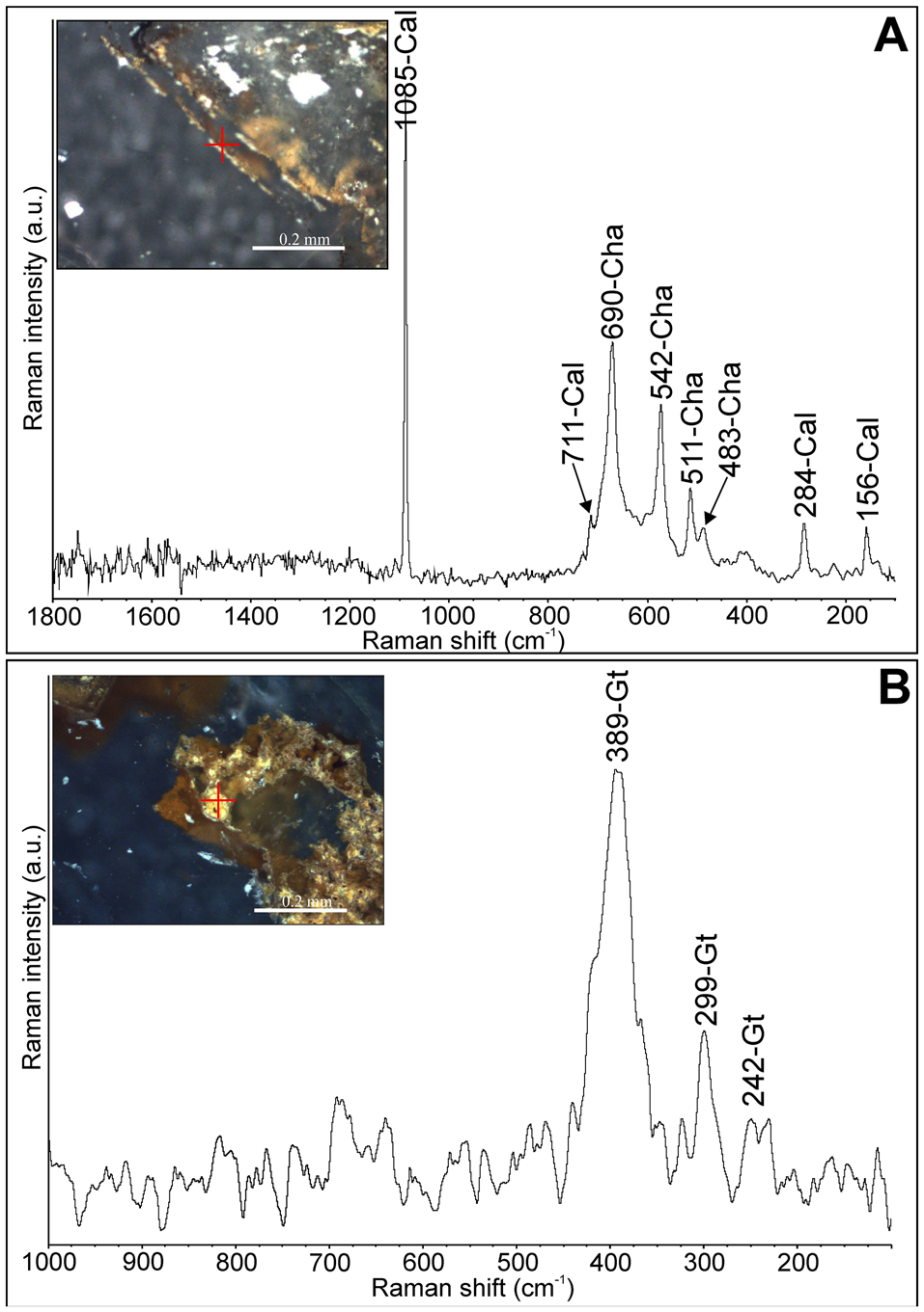
Microphotos of solid inclusions in calcite (Cal) and their Raman spectra: (**A**) chalcophanite (Cha); (**B**) goethite (Gt).

Raman spectroscopy revealed chalcophanite (Fig. 11A) and goethite (Fig. 11B) inclusions hosted in calcite crystals. The marker bands for chalcophanite occur at 483, 511, 542, and 690 cm^−1^, which well correspond with data reported by Julien et al.^32^. Goethite is characterized by Raman bands at 242, 299, 389, and 680 cm^−1^, cf. Das and Henry^33^. The fluid inclusions do not show any admixture of Raman-active gases.

### Stable isotope data

**Figure 12:**
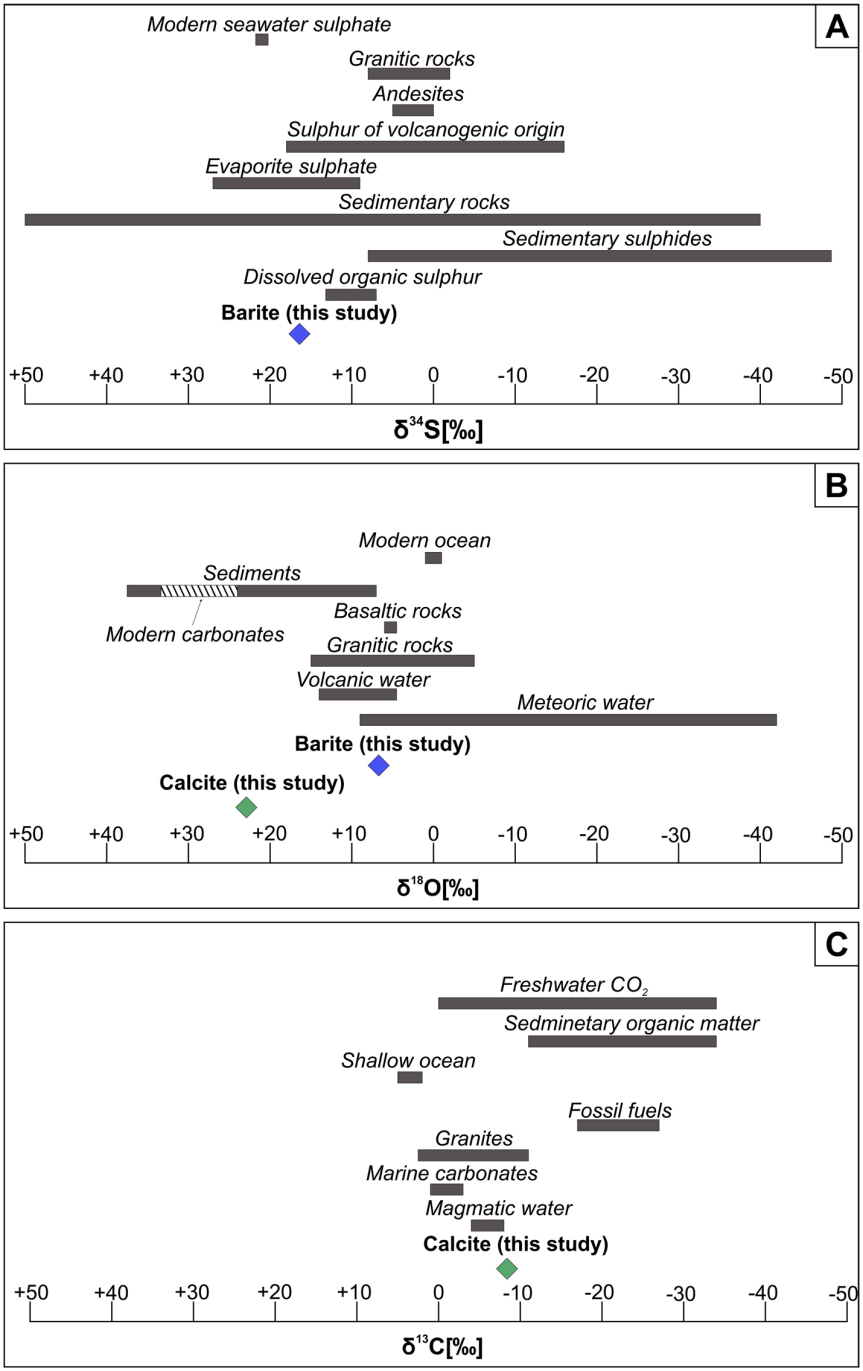
Distribution of δ^34^S (**A**), δ^18^O (**B**), and δ^13^C (**C**) in barite and calcite from Jebel Quichane in relation to various geological reservoirs; data after^48,60–68^.

The sulphur (*δ*^34^S) and oxygen isotope (*δ*^18^O) values of blue barite are measured to be + 16.39‰ (VCDT) and + 6.71‰ (VSMOW), respectively. The coexisting calcite has carbon isotope (*δ*^13^C) values − 8.38‰ (VPDB), and the oxygen isotope (*δ*^18^O) value is + 22.90‰ (VSMOW). The comparison of the obtained results with isotopic data reported from various geological settings is shown in Fig. 12A–C. The sulphur (*δ*^34^S) isotope value of barite not only covers the range of evaporate sulphate and sulphur of volcanogenic origin but also lies close to the range of dissolved organic sulphur. Oxygen isotope (*δ*^18^O) values seem to be quite similar to those reported from i.e. meteoric and volcanic waters. Oxygen (*δ*^18^O) sulphur (*δ*^34^S) isotope values for coexisting calcite are, in turn, consistent with modern carbonates and magmatic water, respectively.

## Discussion

The occurrences as lining vugs in the host skarns implies an epigenetic character of barite from Nador. Paragenetic calcite had probably crystallized before barite as shown by textural relationships between those phases (i.e. the presence of smaller barite crystals on the calcite matrix). Moreover, calcite contains solid inclusions made of chalcophanite and goethite suggesting that calcite-forming fluids were enriching not only in Ca and CO_2_ but also iron and manganese. The spatial distribution of these solid inclusions (concentrating mainly in the core, and diminishing towards the rim) indicates that the content of manganese and iron gradually decreased in the hydrothermal fluids during the crystallization of the host calcite. Previous, experimental works of Shikazono^34^, Kowacz et al^35^, Widanagamage^36^ have just shown that barite features such as morphology, crystal roughness, and crystallinity are related to the parameters of saturation, concentrations of ions, ph and hydrodynamic conditions during its precipitation. Hence, the morphology of barite crystals from Jebel Ouichane in the form of tabular-bladed, well-developed rhomboidal shapes may indicate slow growth rates at a relatively low supersaturation level and under control of a surface reaction precipitation mechanism^34^. The well-formed morphology and relatively large size of gem-quality barite crystals are also caused by the availability of large open spaces as vugs in Fe ore-bearing skarns hosted by Jurassic limestones.

The composition of barite is close to ideal stoichiometric, although Ba is locally substituted by Sr (0.00–0.06 apfu) in the crystal cores due to fluctuations in fluid compositions. In general, Sr shows variations within the individual growth zones since it mainly concentrates in the inner domains of the barite crystals. Such behaviour may reflect diffusion-controlled rates of transport of Sr and Ba^1^. Moreover, chemical zonation in barite may indicate that the very first stage of barite precipitation was characterized by a relatively high growth rate, followed by relatively high supersaturation conditions and rapidly oscillating temperature conditions^37^. It remains consistent with observations of fluid inclusion in barite. The occurrence of densely packed primary liquid inclusions of the first type, forming opaque belts along the growth zones proves episodic rapid crystal growth rate. Occasionally, the dissolution and then recrystallization of barite crystal took place, which is revealed by blurred surfaces marked by some fluid inclusions assemblages (Fig. 4A). Over time, where the crystal zones became wider and wider, the fluid could be less saturated, and environmental conditions have become more stable.

### Conditions of barite precipitation

The predominance of monophase, liquid inclusions in barite is considered as an indicator of low-temperature conditions of crystals growth, presumable 60–70 °C. Similarly, the shape of the Raman spectrum of disordered carbonaceous matter hosted in barite may suggest low-temperature (< 100 °C) thermal activity^31^. This conclusion suggests lower crystallization temperatures than those obtained for other minerals representing a late retrograde stage of mineralization skarn-type deposits, i.e. quartz and calcite, which homogenize under a temperature range of^21^ 250–125 °C.

Due to the fact, that it was impossible to obtain homogenization temperatures of fluid inclusions, the precipitation temperature of barite was estimated using the isotope fractionation–temperature equation proposed by Kusakabe and Chiba^38^, i.e.:

- 10^3^ln α mineral–water = 3.01(10^6^/T^2^) − 7.310^3^ln α mineral–water = 3.01(10^6^/T^2^) − 7.3

where α_barite-water_ is defined by (^18^O/^16^O)barite/(^18^O/^16^O)water, and T is in Kelvin units.

It was assumed that the oxygen isotopic composition of barite-forming fluid corresponds to the value for meteoric waters, i.e. − 7.0‰ (VSMOW)^39^. As a result, the temperature was calculated at 106 °C. However, the data of fluid inclusions indicate that barite formation temperatures were below 100 °C. Thus, the slight discrepancy between temperatures obtained from isotopic composition and data from fluids inclusion could be explained by the presence of strongly-diluted, low-salinity (av. 7.17–7.86 wt% NaCl eq) mineralizing fluid depleted in heavy oxygen isotopes. Assuming lower values of *δ*^18^O for barite-forming fluid (e.g. − 11‰ VSMOW), the calculated temperature would be ~ 70 °C, covering the probable temperature range obtained from fluid inclusion observations. The interpretation of our results stays in agreement with Bouabdellah et al.^21^, who concluded that sulphides and calcite-barite assemblages hosted in skarn of the Ouichane deposit were deposited due to the infiltration of waters of meteoric origin mixed with magmatic-hydrothermal solutions. Much earlier, in the temperature range of 500–400 °C, iron oxides (magnetite-hematite) crystallized^21^.

### Origin of barite mineralization

*δ*^34^S of barite (+ 16.39‰VCDT) is used as a premise for the nature of Ba and SO_4_-rich fluids. Such a high isotopic value is probably associated with the migration of barite-forming solutions in rocks enriched in organic sulphur^40–42^, as it is generally accepted that organosulphur compounds are enriched in heavy S isotope relative to the coexisting sulphides such as pyrite^43^. The sulphur isotopic composition of the barite is not only consistent with the values adopted for evaporites of the Mesozoic age^44^ but also covers the range of hydrothermal sulphates described by Jurkowić et al.^45^. The *δ*^18^O (+ 6.71 VSMOW) is characteristic of meteoric waters^46^, but also fluids related to volcanic activity (Fig. 12B). Hence, the mixing mechanism of meteoric waters with hydrothermal fluids is responsible for barite precipitation.

The *δ*^13^C depletion in calcite (-8.38 VPDB) points to the magmatic affinity of the mineral-forming CO_2_-bearing fluids originating from i.e. igneous country rocks^47,48^ – see Fig. 12C. On the contrary, *δ*^18^O (+ 22.90 VSMOW) is characteristic of marine solutions (Fig. 12B) and Mesozoic sediments^44,49^. The discrepancy between *δ*^18^O of calcite (+ 22.90) and barite (+ 6.71) may indicate that calcite formed early via dissolution and decarbonation of pre-existing limestones by magmatic-related waters, whereas barite-forming parental fluids were derived later from variable sources.

Barite precipitation is typically induced by mixing of SO_4_^2—^rich fluid with Ba-rich solutions. The low solubility of this sulphate in hydrothermal conditions indicates that both main barite components were not transported together in one fluid^50^. To determine the source of sulphate necessary for barite crystallization we initially assume the following scenarios including (1) direct dissolution of evaporites, (2) oxidation of sulphide minerals, or (3) mineralization of organic substance^51^. In Nador, evaporite deposits are not known from the rock sequences in that area and thus cannot be considered as a possible source for SO_4_-rich fluids. Nevertheless, the abundant pyrite was found in the Jurassic-Cretaceous limestones from the study area^21^. Hence, the production of sulphate as a result of sulphide oxidation might be possible in this geological setting. On the other hand, both pyrite and barite may form contemporaneously, as a result of low-temperature hydrothermal activity. Finally, the enrichment in *δ*^34^S might suggest an organic origin related to the thermal decomposition of organosulphur in the limestones^42,51^. Alternatively, the relationship between both δ^34^S and δ^18^O data of barite provides evidence for the mixing of HSO_4_ and SO_4_^2-^ derived from magmatic-hydrothermal SO_2_ with sulphates from the oxidation of H_2_S near the earth surface (steam-heated) conditions^6,50,52^. In a steam-heated meteoric groundwater environment, H_2_S originally derived from degassing magma or via the disproportionation reaction of magmatic SO_2_ may be oxidized by atmospheric oxygen following reaction^6^ H_2_S + 2O_2_ = H_2_SO_4_ . This reaction takes place at or above the water table, where the temperature does not exceed^53^ 100 °C. In such conditions, the acid fluid may leach and dissolve primary Fe-bearing phases to produce pyrite or some sulphate minerals^54^. On the other hand, the Ba-bearing solutions, needed for barite formation, were probably also charged in in Sr, Al, K, and Na. They might have been derived from the alteration of feldspars and carbonate minerals, being components of both the sedimentary and igneous rocks (diorites, granites, andesites) found in the vicinity of Jebel Ouichane.

## Conclusions

1. Gem-quality light-blue barite from Jebel Ouichane (Nador) displays simple and homogenous chemical composition except for local substitution of Ba by Sr (up to 2.59 wt% of SrO) in the inner regions of particular crystals. The abundance of monophase, liquid inclusions, supported by the presence of poorly-ordered organic matter, suggests that barite crystallized from low-temperature (< 100 °C) and low-salinity fluids enriched in light oxygen isotope.
2. Stable isotope data for barite (δ^34^S = + 16.39‰, δ^16^O + 6.71‰) indicate a mixing episode and reflect two distinct sources of sulphate, i.e. steam-heated (near-surface) and strongly diluted meteoric water that reacted with ascending magmatic-hydrothermal fluid related to the Miocene porhyric intrusion. The enrichment of barite in heavy S isotope might also suggest that some amounts of sulphur sourced from oxidation of pyrite and/or decomposition of limestone-hosted organosulphur compounds.
3. High δ^18^O values coupled with low δ^13^C values (+ 22.90 and − 8.38‰, respectively) of the calcite associated with barite may reflect the decarbonation and/or dissolution of pre-existing sedimentary carbonates, during metasomatic interactions between magmatic-related fluids and Jurassic limestones found in the study area, and the subsequent precipitation of calcite in vugs.
4. The probable sequence of crystallization in Ouichane iron deposit could proceed as follows: (1) iron oxides mineralization within skarns, (2) deposition of calcite abundant in iron and manganese solid inclusions, (3) formation of gem-quality barite devoid of any ore-bearing inclusions.

## Methods

Barite and coexisting calcite samples were investigated in this work using microscopic, spectroscopic, micro-chemical, and isotopic methods. The presence of fluid inclusions was described using optical microscopy and supported by microthermometry. The maps of element distribution in barite clusters were obtained with micro X-ray Fluorescence. The detailed geochemistry of barite in the micro-region was obtained with electron microprobe analyses. The chemical data were correlated with the results of Raman micro-spectroscopy. The temperature of crystallization of barite and was additionally estimated with oxygen isotope analyses. The map showing simplified geological map of the region was created using CorelDRAW X6.

### Optical microscopy

The iron ore, which hosts barite crystals, was analysed with Olympus BX 51 polarizing microscope with a magnification ranging from 40 × to 400 × . The observations were conducted using both transmitted and reflected light modes. The photomicrographs were acquired using an Olympus DP12 digital camera equipped with the Analysis software. The wafers and thin sections of barite and calcite crystals were examined with both Motic SMZ168 binocular with a magnification range of 0.5 × , 1 × , 2 × , 3 × , 4 × , 5 × and Motic BA310Pol polarizing microscope with objectives of 4 × , 10 × , 40 × , and 60 × to provide the general description of various kinds of inclusions in both minerals.

### Fluid inclusion analysis

Barite-hosted fluid inclusions were analysed on double-polished wafers (0.2 mm thick) by using both Linkam FTIR600 stage mounted on the ZEISS AxioScope A1 microscope with magnification objectives of 10 × , 50 × , and 100 × , equipped with QImaging Micro Publisher 5.0 RTV camera and Linkam THMSG600 stage mounted on the Olympus microscope BX53 with magnification objectives of 5 × , 10 × , 20 × , 50 × , and 100 × equipped with Olympus UC90 camera. The first piece of equipment was available for studies at the Slovak Academy of Sciences in Banska Bystrica, the second at the Faculty of Geology, Geophysics and Environmental Protection AGH University of Science and Technology in Krakow. In both cases, the calibration of the stage was carried out using natural inclusions of pure CO_2_, and chemical compounds with known temperatures of phase transitions. The fluid inclusions were subjected to temperatures in the range from − 180 to + 250 °C. The heating runs were made at the rate of 5 °C/min until the final ice melting temperature was approaching.

Samples devoid of two-phase inclusions at room temperature were previously cooled at 5 °C for 24 h in the fridge and at 0.1 °C for several minutes in the freezing-heating stage to induce the vapor nucleation.

### X-ray fluorescence spectrometry

The Energy-dispersive micro X-ray Fluorescence Spectrometry analysis was performed using the M4 TORNADO (Bruker) spectrometer. The maps of elements distribution were obtained from the selected area of 29 mm × 18.4 mm within a polished crystals aggregate. The excitation current (Rh anode) was 600 μA at 50 kV. The analyses were carried out in a vacuum of 20 mbar, the distance between the two measurement points was 15 μm, at a speed of 20 ms/pixel. The SDD detector that collects the fluorescent signal has an active area of 30 mm2 and a spectral resolution of 145 eV. Elements concentration was computed by the fundamental parameters method.

### Electron microprobe microanalyses

EPMA analyses were performed with a JEOL Super Probe JXA-8230 operating in a wavelength-dispersive (WDS) mode under the following conditions: an accelerating voltage of 15 kV, a beam current of 20 nA, beam size of 2 µm, a peak count-time of 20 s, and a background time of 10 s. The EMPA standards, analytical lines, diffracting crystals, and mean detection limits were as follows: Na- albite_SPI (Kα, TAP, 478 ppm), Sr-celestine (Lα, PETH, 736 ppm), Ca-diopside (Kα, PETL, 278 ppm), S-anhydrite (K, PETJ, 666 ppm), Ba-barite (Lα, PETL, 741 ppm). The other analysed elements such as Al, Mg, Mn, Fe, Cl, Pb, L, Zn, Cu were under the detection limit of the method. The JEOL ZAF procedure was used for the matrix correction of the raw data.

### Raman micro-spectroscopy

Raman spectra of barite, calcite, and solid inclusions hosted in them were recorded with a Thermo Scientific DXR Raman microscope featuring 10x, 50x, and 100 × magnification objectives. The samples were excited with a 532 nm high-power laser. Laser power was from 5 to 10 mW, the exposure time was 3 s, the number of exposures—10 times. The laser focus diameter was approximately 2.1–0.7 mm. The spectra were corrected for background by a method of a sextic polynomial using Omnic software. Raman analyses were made both on clean cleavage surfaces and doubly polished wafers. Raman studies were performed in the same analytical spots of barite, for which chemical analyses were carried out using the EMPA method to trace the differences in the position of individual Raman bands in the points differing in the Sr contents.

Raman spectra from fluid inclusions hosted in barite were collected with (1) the Renishaw inVia spectrometer, connected to a Leica microscope, and (2) the Thermo Scientific Nicolet NXR 9650 FT-Raman spectrometer equipped with a Micro-Stage Microscope. In the first equipment, the samples were excited with a 1064 nm line of the Nd:YAG laser applying the power of 500 mW. The resolution parameter was set to 4 cm^−1^. There were accumulated 100 scans. For the measurements of the Raman spectra exciting sample with the 514.5 nm line of Ar^+^ ion Modu-Laser the Renishaw inVia spectrometer connected to a Leica microscope was used. The laser beam was focused by 100 × magnifying, a high numerical aperture (NA = 0.80) top-class Leica objective for standard applications. Raman light was dispersed by a diffraction grating of 2400 l/mm. Laser power was kept rather low, c.a. 1–3 mW at the sample, the number of accumulations was equal to 4.

### Isotope analyses

The isotope ratios of barite (*δ*^34^S and *δ*^18^O) were determined by measuring the isotopic composition of SO_2_ and CO_2_ gases on a dual-inlet and triple collector mass spectrometer. Sulphur in the form of SO_2_ gas was quantitatively extracted from the BaSO_4_ sample by thermal decomposition at 850 °C in a Cu boat in the presence of Na_2_PO_4_ reagent^55,56^. CO_2_ gas was prepared by graphite reduction with the conversion of CO to CO_2_ by glow discharge^57^. Nearly quantitative CO to CO_2_ conversion was attained using a magnetic field in the conversion unit^58^. The rough delta values were normalized to the Vienna-Canyon Diablo Troilite (VCDT) and the Vienna Standard Mean Ocean Water (VSMOW) standards by analysis of the SO_2_ and CO_2_ raw isotopic ratios prepared from the NBS-127 standard, for which we assumed *δ*^34^S = 21.17‰^55^ and *δ*^18^O = 8.73‰^58^.

For the accompanying calcite, the *δ*^13^C and *δ*^18^O values were determined as well. CO_2_ gas was extracted from calcite at 25 °C by reaction with H_3_PO_4_^59^ and measured on an isotope-ratio mass spectrometer with a dual-inlet system. The standard deviations of measurements for the NBS19 international standard were better than 0.1‰. Delta values were normalized to the Vienna Pee-Dee Belemnite (VPDB).
